# Developing an AI-Assisted Tool That Identifies Patients With Multimorbidity and Complex Polypharmacy to Improve the Process of Medication Reviews: Qualitative Interview and Focus Group Study

**DOI:** 10.2196/74304

**Published:** 2026-01-08

**Authors:** Aseel S Abuzour, Samantha A Wilson, Alan A Woodall, Frances S Mair, Asra Aslam, Andrew Clegg, Eduard Shantsila, Mark Gabbay, Michael Abaho, Danushka Bollegala, Harriet Cant, Alan Griffiths, Layik Hama, Gary Leeming, Emma Lo, Simon Maskell, Maurice O'Connell, Olusegun Popoola, Sam Relton, Roy A Ruddle, Pieta Schofield, Matthew Sperrin, Tjeerd Van Staa, Iain Buchan, Lauren E Walker

**Affiliations:** 1 Academic Unit for Ageing & Stroke Research University of Leeds Leeds United Kingdom; 2 School of Medicine Faculty of Medicine and Health University of Leeds Leeds United Kingdom; 3 Institute of Population Health University of Liverpool Liverpool United Kingdom; 4 Directorate of Mental Health and Learning Disabilities Powys Teaching Health Board Bronllys United Kingdom; 5 School of Health and Wellbeing General Practice and Primary Care University of Glasgow Glasgow United Kingdom; 6 Leeds Institute for Data Analytics University of Leeds Leeds United Kingdom; 7 Department of Computer Science University of Liverpool Liverpool United Kingdom; 8 Division of Informatics, Imaging & Data Science University of Manchester Manchester United Kingdom; 9 NIHR Applied Research Collaboration North West Coast Liverpool United Kingdom; 10 School of Computer Science University of Leeds Leeds United Kingdom; 11 Department of Electrical Engineering and Electronics University of Liverpool Liverpool United Kingdom; 12 Mersey Care NHS Foundation Trust Liverpool United Kingdom; 13 Centre for Experimental Therapeutics University of Liverpool Liverpool United Kingdom; 14 Liverpool University Hospitals NHS Foundation Trust Liverpool United Kingdom

**Keywords:** structured medication reviews, medicine optimization, health technology, risk stratification, artificial intelligence, AI

## Abstract

**Background:**

Structured medication reviews (SMRs) are an essential component of medication optimization, especially for patients with multimorbidity and polypharmacy. However, the process remains challenging due to the complexities of patient data, time constraints, and the need for coordination among health care professionals (HCPs). This study explores HCPs’ perspectives on the integration of artificial intelligence (AI)–assisted tools to enhance the SMR process, with a focus on the potential benefits of and barriers to adoption.

**Objective:**

This study aims to identify the key user requirements for AI-assisted tools to improve the efficiency and effectiveness of SMRs, specifically for patients with multimorbidity, complex polypharmacy, and frailty.

**Methods:**

A qualitative study was conducted involving focus groups and semistructured interviews with HCPs and patients in the United Kingdom. Participants included physicians, pharmacists, clinical pharmacologists, psychiatrists from primary and secondary care, a policy maker, and patients with multimorbidity. Data were analyzed using a hybrid inductive and deductive thematic analysis approach to identify themes related to AI-assisted tool functionality, workflow integration, user-interface visualization, and usability in the SMR process.

**Results:**

Four major themes emerged from the analysis: innovative AI potential, optimizing electronic patient record visualization, functionality of the AI tool for SMRs, and facilitators of and barriers to AI tool implementation. HCPs identified the potential of AI to support patient identification and prioritizing those at risk of medication-related harm. AI-assisted tools were viewed as essential in detecting prescribing gaps, drug interactions, and patient risk trajectories over time. Participants emphasized the importance of presenting patient data in an intuitive format, with a patient interface for shared decision-making. Suggestions included color-coding blood results, highlighting critical medication reviews, and providing timelines of patient medical histories. HCPs stressed the need for AI tools to integrate seamlessly with existing electronic patient record systems and provide actionable insights without overwhelming users with excessive notifications or “pop-up” alerts. Factors influencing the uptake of AI-assisted tools included the need for user-friendly design, evidence of tool effectiveness (though some were skeptical about the predictive accuracy of AI models), and addressing concerns around digital exclusion.

**Conclusions:**

The findings highlight the potential for AI-assisted tools to streamline and optimize the SMR process, particularly for patients with multimorbidity and complex polypharmacy. However, successful implementation depends on addressing concerns related to workflow integration, user acceptance, and evidence of effectiveness. User-centered design is crucial to ensure that AI-assisted tools support HCPs in delivering high-quality, patient-centered care while minimizing cognitive overload and alert fatigue.

## Introduction

### Background

The growing prevalence of multiple long-term conditions and complex polypharmacy among older adults poses significant challenges for health care systems globally. Structured medication reviews (SMRs) are a key clinical intervention, approved by the National Institute for Health and Care Excellence (NICE), designed to facilitate shared decision-making between clinicians and patients, optimize prescribing, and reduce medication-related harm in patients at high risk who are experiencing problematic polypharmacy [1,2].

General practitioners (GPs), pharmacists, and advanced nurse practitioners who meet training criteria can conduct SMRs. As a commissioned service, the prevailing expectation is for clinical pharmacists within primary care networks (PCNs) to proactively identify patients suitable for an SMR and conduct these reviews [3]. However, it is increasingly recognized that effective SMRs are difficult to implement clinically due to time pressures, fragmented clinical records, and the cognitive burden placed on clinicians when trying to assimilate information from various different sources in order to make shared, person-centered decisions [4].

Currently, in the United Kingdom, a few artificial intelligence (AI)–assisted tools are available to help health care professionals (HCPs) prioritize patients for SMRs. Tools available are usually based on predefined conditions or medications; the examples include Ardens Search [5] and Proactive Register Management Diabetes [4,6]. Prescribing safety indicators have also been used as a technology-based intervention to identify potentially inappropriate prescribing to reduce the number of patients at risk of hazardous prescribing [7,8]. However, primary care–embedded clinical decision support systems (CDSSs), such as audit and feedback tools, are often limited by data supply [9]. Emerging digital technologies, including AI, offer opportunities to enhance the efficiency and effectiveness of the SMR process through automation of routine tasks, rapid data extraction and synthesis, and highlighting clinical risks to support decision-making. However, the integration of AI into clinical workflows is in its infancy, and questions exist about its accuracy, clinical utility, usability, and trust. These implementation barriers are currently unexplored.

This study is part of a larger DynAIRx (AI for dynamic prescribing optimization and care integration in multimorbidity) project. Our research to date has highlighted the time-intensive nature of SMRs and the lack of AI-assisted tools to efficiently identify and prioritize patients [4]. Findings emphasized the need for an AI-assisted tool to identify, prioritize, and reduce the time needed to understand the patient journey in order to optimize medicines appropriately and reduce the risk of potential harm from medicines [4]. DynAIRx involves developing novel AI-assisted approaches to improve the efficiency of SMRs. The planned DynAIRx tool will comprise 4 main components: stratification of patients, clinical trial emulation to understand real-world risk of deprescribing, patient journey visualization through interactive timelines, and a knowledge support system integrating individualized patient risks to support decision-making. The deep learning AI component of this is to develop a tool to stratify patients most in need of an SMR. The DynAIRx stratification tool will compare 2 main approaches to identifying which patients are most at risk of medication-related harm: investigating the trade-off between model performance and explainability in the SMR context. First, a simple logistic regression model not only gives a baseline performance level to assess the AI-based approach but also is clearly explainable and technically feasible to implement within clinical systems, such as EMIS and SystmOne. Second, a novel approach based on graph neural networks will be used to incorporate the sequence and timing of clinical events into predictions. This is likely to offer superior performance but will be difficult to explain and implement.

Large language models (LLMs), such as ChatGPT (OpenAI), are breaking new ground as an adjunct to support clinical decision-making. In radiological decision-making, ChatGPT recently showed impressive accuracy in the appropriate identification of imaging to support breast cancer screening [10]. There are several proof-of-concept AI-assisted tools in development to support complex polypharmacy. For example, the approach based on discriminator-enhanced encoder-decoder architecture for accurate prediction of adverse effects in polypharmacy is an AI model developed to predict adverse drug-drug interactions [11]. However, its effectiveness in a clinical setting has not yet been attempted. A new LLM based on retrieval-augmented generation has been developed to support pharmacists in identifying medicine errors [12]. User testing has been undertaken with simulation of complex scenarios and a multidisciplinary expert panel; however, true workforce implementation is still to be undertaken. Drug GPT is a specialized proprietary LLM tool for predicting medication safety events, developed by Oxford’s AI for Healthcare Lab. It initially garnered popular attention upon the release of the preprint in 2023, including a review in the *Guardian* [13]. However, the preprint was subsequently removed by the authors, and its route to clinical implementation remains unclear [14]. Despite rapid progress, there remains limited understanding of what HCPs and patients actually need from such tools to support SMRs and, importantly, how to embed them into routine clinical practice, particularly in the context of multimorbidity and complex care.

While not all components of the DynAIRx polypharmacy tool will be AI assisted, the field of AI-assisted health technology is rapidly advancing. Consequently, it is critical to understand the user requirements for AI-assisted technologies now, as it may in fact be the case that simple, non-AI solutions can address the challenge in a straightforward and explainable way. Therefore, the health care sector is at a critical juncture when it comes to understanding end-user requirements for medication support.

### This Study

This study aimed to explore the perspectives of both HCPs and patients on the potential role of AI in supporting SMRs, with a focus on identifying the core user requirements, anticipated benefits, and key barriers to implementation.

## Methods

### Participants and Recruitment

This study sought to recruit HCPs or management professionals from UK primary care (community based) and secondary care (hospital services) settings, for whom reviews of prescription medications form a routine part of clinical workload. Participants included those working in general practice; secondary care hospital services (geriatric medicine, clinical pharmacology, falls clinics, and mental health practitioners); clinical commissioning, service management (practice managers); and pharmacists, including PCN pharmacists who conduct SMRs across multiple GP practices. Patient participants included (1) those with mental and physical comorbidities, (2) those with complex multimorbidities, and (3) older people with frailty. In addition, patient and carer representatives from these 3 key multimorbidity groups were recruited, comprising adults aged >18 years with or caring for someone with multimorbidity (4 or more), coexisting mental and physical health problems, ≥10 or more prescribed medications, or frailty. Patient participants self-identified as not digitally engaged. As the General Data Protection Regulation was not required, we did not collect demographic data from patient participants.

Purposive sampling identified potential HCP participants actively involved in medicine optimization services through the researcher’s clinical and professional networks. Snowball sampling, where current participants referred others, helped identify contacts through existing service providers and advertisements in GP forums and at national events related to clinical polypharmacy research. Patient representatives were recruited purposively via advertisements through the National Institute for Health and Care Research Applied Research Collaboration public advisor networks and research databases at the researcher’s host institutions.

### Ethical Considerations

The Newcastle North Tyneside Research Ethics Committee (22/NE/0088) granted ethical approval for the DynAIRx study. Written consent was obtained before participation, and withdrawal of consent was permitted at any stage, including after data collection. Audio recordings were transcribed verbatim, anonymized to remove any potentially identifiable information, and assigned participant codes before recordings were subsequently deleted. All data were stored on secure servers in accordance with data protection regulations. Participants received modest compensation in the form of a voucher to acknowledge their time and contribution, consistent with ethical guidance. No participants withdrew consent for the use of their data in this study. Sessions were conducted in person and online (via Microsoft Teams), lasting from 49 to 109 minutes. Data collection and analysis occurred concurrently, adhering to the COREQ (Consolidated Criteria for Reporting Qualitative Research) checklist for comprehensive reporting (Multimedia Appendix 1).

### Data Collection

**Figure 1:**
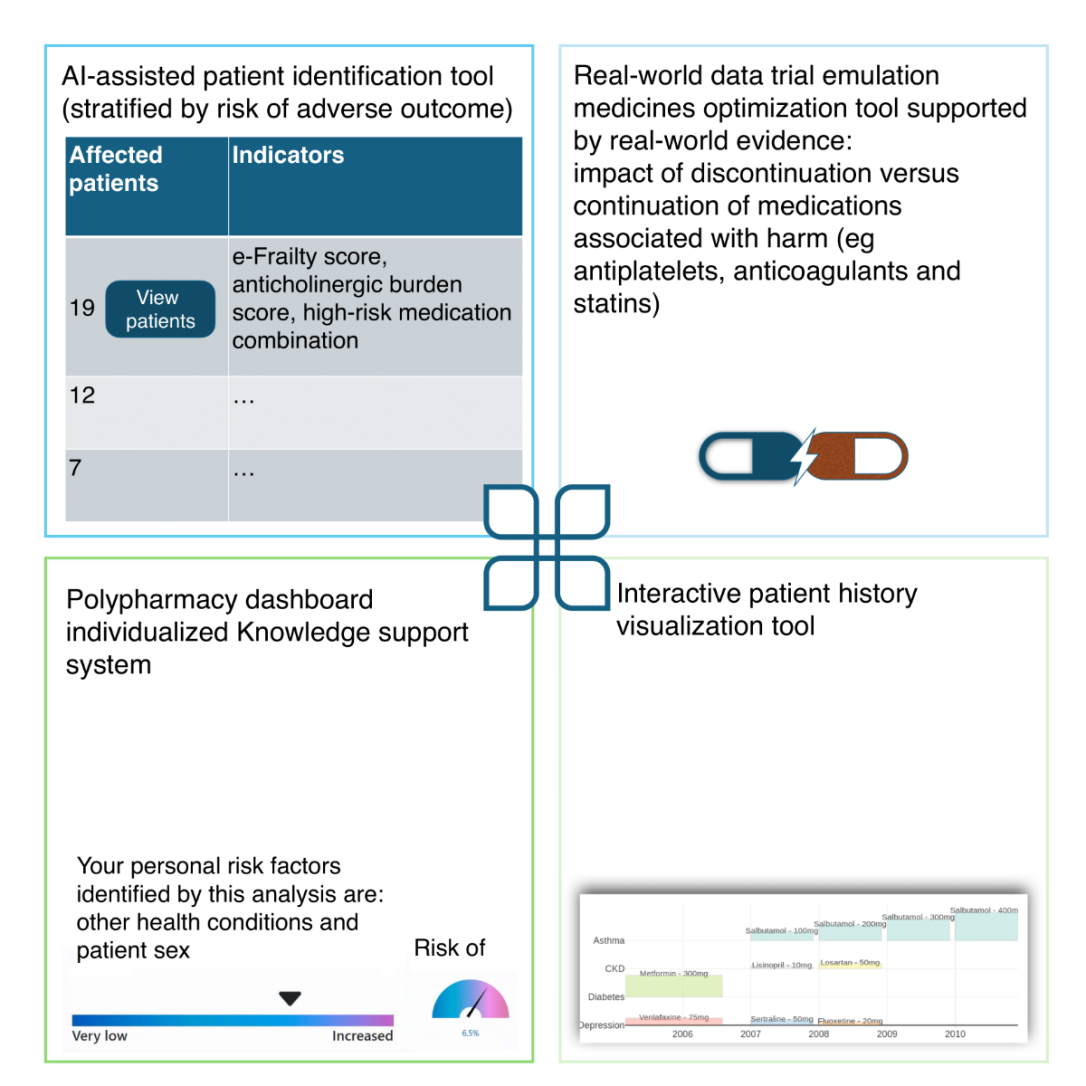
Visual representation of proposed components of an artificial intelligence (AI)–assisted tool (the DynAIRx tool [AI for dynamic prescribing optimization and care integration in multimorbidity]).

Data collection occurred from November 2022 to November 2023. Focus groups (FGs) and semistructured interviews were conducted to gather participants’ views. Patient participants were involved in FGs to discuss their shared experiences, while FGs and individual interviews were conducted with HCPs to accommodate time constraints. A semistructured topic guide was developed collaboratively by the research team, informed by existing literature and expert input. The topic guide focused on key elements relevant to the study aims and reflected the literature and practical insights from clinical practice. This guide was used consistently across both FGs and interviews. Interviews were conducted to complement purposive sampling and address any gaps in representation. These took place either before FGs, to inform key discussion areas, or alongside them for participants unable to attend a group session. FG topic guides and interview schedules were developed and refined by the clinical members of the research team (LEW, AA, AAW, FSM, and AG) and tailored to HCP and patient groups. The topic guides explored the current challenges and limitations in the SMR process that existing advancements in AI and machine learning (ML) can address and the essential components for a user-friendly prescriber feedback system. We also asked participants questions to identify the key components and functionalities needed in a prescriber feedback system to ensure it is useful and user-friendly. Figure 1 depicts the visual representation of the proposed components of an AI-assisted tool (the DynAIRx tool).

### Data Analysis

Data were analyzed thematically, with coding independently conducted by researchers (AA and SAW). Researchers read transcripts to familiarize themselves with the data. Initial coding, guided by inductive reasoning, was conducted by AA and SAW, who collated and examined codes to identify themes. The interview and FG transcripts were coded concurrently to capture both individual and group perspectives. The multidisciplinary coding team (AA, SAW, LEW, AAW, and FSM), comprising clinicians and researchers, engaged in regular coding clinics in a reflexive practice to ensure rigorous and transparent qualitative analysis. These sessions were used to discuss emerging codes, refine codes, and assess data saturation. Discrepancies were resolved through discussion with the wider team of researchers and clinicians, ensuring diverse perspectives informed theme development and interpretation. This reflexive approach balanced interpretations and mitigated biases, grounding the analysis in participants’ narratives. Themes were defined and supported by quotes, with detailed notes maintained to ensure analytic rigor and plausibility. The dataset underwent hybrid inductive and deductive thematic analysis, with iterative revisions of codes and themes. Data saturation was defined as the point at which no new themes or subthemes emerged from the data concerning the already existing themes, which were richly supported by the data. During coding clinics, the research team compared newly coded transcripts against the developing codes to assess whether additional data contributed to novel insights. Recruitment ceased once all team members agreed that no new codes or meanings emerged from successive transcripts, and the thematic structure was considered sufficiently rich to address the study aims and indicate thematic saturation.

## Results

### Overview

In total, 6 FGs with HCPs (n=21) and 3 FGs with patients (n=13) were conducted (Table 1). A further 5 semistructured interviews with HCPs took place (Table 2). The interviews were undertaken to explore topics in greater depth and address any gaps in purposive sampling. Moreover, the number of participants in each FG differed based on HCPs’ availability. However, this did not impact data analysis or saturation, as all HCPs undertook SMRs and expressed their views and requirements for an AI-assisted tool to allow the SMR process to be more efficient and effective.

**Table 1:** Participant type and the number of participants who took part in the focus groups (FGs; n=34).

FGs	Participants, n (%)
General practitioner FG1	2 (6)
General practitioner FG2	6 (18)
Pharmacist^a^ FG1	3 (9)
Pharmacist FG2	5 (15)
Clinical pharmacologist FG1	3 (9)
Psychiatrist FG (mix of secondary care and prison care)	2 (6)
Patient FG comprising individuals with mental and physical health comorbidities for whom prescribing for mental health improvement could lead to adverse physical health consequences	6 (18)
Patient FG comprising those with complex multimorbidity (≥4 long-term health conditions and taking ≥10 drugs)	4 (12)
Patient FG comprising older people with frailty who were at a high risk of adverse outcomes	3 (9)

^a^Mix of primary and secondary care pharmacists.

**Table 2:** Participant type and the number of participants who took part in the semistructured interviews (n=5).

Participant	Interviews, n (%)
Primary care pharmacist	1 (20)
Secondary care pharmacist	1 (20)
Policy maker	1 (20)
Secondary care psychiatrist	1 (20)
Postgraduate GP^a^ trainee	1 (20)

^a^GP: general practitioner.

HCPs conducted SMRs either proactively or reactively, depending on staff capacity, organizational contracts, and practice size. The presence of a PCN pharmacist facilitated proactive SMRs by ensuring that patients meeting directed enhanced service requirements were identified and invited for an SMR. In contrast, GPs and secondary care clinicians often conducted opportunistic medication reviews. Regardless of how patients were identified for a medication review, HCPs described the significant preparation time required to gather and interpret patient information, citing the lack of efficient methods to identify patients at risk of medication-related harm who would benefit most from an SMR [4]. This prompted discussion on how AI approaches could be used to improve the SMR process, including the potential barriers to the uptake and use of AI-assisted tools to support SMRs.

The following 4 overarching themes were developed from the analysis:

1. Innovative AI potential
2. Optimizing electronic patient record (EPR) visualization
3. Functionality of the AI tool for SMRs
4. Facilitators of and barriers to AI tool implementation

Innovative AI potential referred to the emerging possibilities and future impacts of applying AI technologies in health care contexts. Optimizing EPR visualization concerned the enhancement of clarity, usability, and accessibility of clinical information presented within EPR systems. Functionality of the AI tool for SMRs examined how the AI-assisted tool operated and supported the delivery of SMRs. Finally, facilitators of and barriers to AI tool implementation encompassed the organizational, technical, and human factors that influenced the successful integration and effective use of AI-assisted tools in clinical practice.

These themes were not entirely discrete; they reflected interrelated aspects of participants’ experiences and perspectives on AI integration in clinical practice, with points of overlap and influence between them. Aspects, such as system usability, functionality, and perceived potential, often interact within the broader context of AI-assisted tools in health care. Participants’ perceptions and responses varied according to their professional or personal role (eg, GP, pharmacist, psychiatrist, and patient), highlighting the need for AI-assisted tools to be sufficiently adaptable to address the differing needs of key stakeholder groups.

### Innovative AI Potential

Participants expressed their views on the potential utility and advantage of AI in identifying patients at risk of medication-related harm or those who might benefit most from an SMR. HCPs emphasized the need for a tool capable of comprehensively searching EPRs to identify patients with complex multimorbidity. Such a tool should dynamically adjust search outcomes in real time, prioritizing patients who require immediate SMRs.

There was a desire to see AI-assisted tools that could learn autonomously from the historical health care record to identify which factors are contributing to potential medication-related harm. Participants showed great interest in the incorporation of AI with a health care tool to automate tasks and reduce delays in risk prediction. In addition, participants wanted these tools to show patients’ medical history in a holistic way using AI capabilities and have the AI be explainable to understand why and how the patient was triggered for a medication review:

If you had a funky IT program that looked at medication, looked at what other stuff was happening, looked at, you know, bloods, these are patients that I’m really worried about. So, you know you’re talking about machine learning in your project, one of the things, you know, I think will be really interesting to do would be to actually ask the computer what the predictors for certain harms are. We know patients when they fall for multiple reasons, it’s not just medicines, but actually wouldn’t it be good to see that these are the key circumstances that patients fall under, and then if those circumstances ever happened that patient would be, you know, triggered for a review.

Policy maker; interview

It could potentially work in real time as well...having something which is live so constantly producing the order of patients who you should be reviewing based on, I don’t know a patient might have been discharged from hospital last night that patient might become a bit more high risk and therefore it needs to review earlier. So it flags upon our systems as a, you know, using the AI that this patient will probably need to review in the next four or five days.

Pharmacist 2; interview

Leveraging data analytics with ML was viewed as an opportunity to flag patients on complex medication regimens by assessing their health records and prioritizing those patients at risk of medication-related harm. Moreover, aligning patient risk levels with the GP practices staff capacity within PCNs would ensure that those who need immediate attention are seen promptly:

That is a real issue for us. It’s a real issue for practice actually. So this is why I think the tools have to be a bit more cleverer than just generating, you know, we can generate a list of patients today and that happens, and PCNs at the moment essentially do that, but what you have to do is almost match the list that’s generated to the capacity of the build this so you can, the practice has to say that across my PCN I’ve got, you know, 100 appointments a week to deal with these sort of patients then the tool has to generate that...People would not switch it on if they felt that it could generate lots of patients you would not then see.

Policy maker; interview

The development of an AI-assisted tool to support the SMR process prompted discussions on how ML tools could predict risk, identify prescribing gaps, highlight lifestyle and family history risk predictors, and detect potential adverse drug reactions. Advanced digital health tools with AI-assisted features and data analytics could enhance patient engagement by enabling holistic discussions about the patient’s risk trajectories and how their medicines can be optimized to reduce any medication-related risks:

There used to be a tool, I think it was developed in Australia or New Zealand, but basically it showed a graph of heart disease and trajectory towards symptoms. And you could have a discussion with patients and you can say well look, if we bring your blood pressure down by this much then this is your trajectory...if we stop you smoking, then this is your trajectory. If you develop diabetes then this is your trajectory, and that was probably the single most powerful tool I had to convince people to optimize things like blood pressure or cholesterol reduction.

Participant 1; GP FG1

I’m sure that having an AI trawl through drug prescribing gaps would tell us quite a lot about medication that may not be taken when it’s supposed to have been. We kind of think about that in terms of people misusing analgesics but actually for the elderly population they’ll very often just order it because they don’t know how to tell us they don’t like it or they don’t want to take it anymore because they’re very much of the mindset that doctor knows best and they don’t want the conflict. But they’ll very often forget to order medication and that can be a giveaway.

Participant 5; GP FG1

I’m thinking of an example where someone has attended an appointment and mentioned that they think a drug is causing a side effect for them, I would imagine that someone would document experiencing this? Being able to see that would be very helpful to try and further add to why things might have been stopped or why a patient might have stopped taking them and maybe not told anyone.

Participant 2; pharmacist FG1

We did a piece of work recently on familial hypercholesterolaemia and pulled up a lot of patients we didn’t realize had family history of massive cholesterol levels and they hadn’t realized it was potentially hereditary.

Participant 5; pharmacist FG2

### Optimizing EPR Visualization

Participants pointed to the challenges associated with the time required to gather and interpret a patient’s medical history, emphasizing the need for an AI-assisted tool that optimizes the presentation of relevant information within the EPR. This included reorganizing readily available data to provide a clearer view of the patient’s medical history and social circumstances to produce accessible visualizations of the medication timelines, including what medications were prescribed for what condition. Several participants suggested displaying the patient’s medical history in a timeline format, detailing key events, such as medication initiation, titration, or discontinuation, diagnosis dates, and recent relevant blood test results:

So something pictorially, which helps represent the information in a clearer way, I suppose. Yeah, maybe more longitudinal kind of...And representation of say, when medications were started and titrated up and previous medications, when they were brought in and when there were stopped.

Psychiatrist; interview

I suppose my top 5 would be: something that highlights previous courses of the same type of medication or the same class, so, for example, if I type in depression as a code, I want an automatic list of every antidepressant they’ve been on previously and how long they’ve been on it and which ones they haven’t had, even the new ones that are coming on line. I want a list of when they had prednisolone last if they have chronic lung disease, [and] how many courses in the last year they’ve had without searching for it.

Participant 1; GP FG1

**Figure 2:**
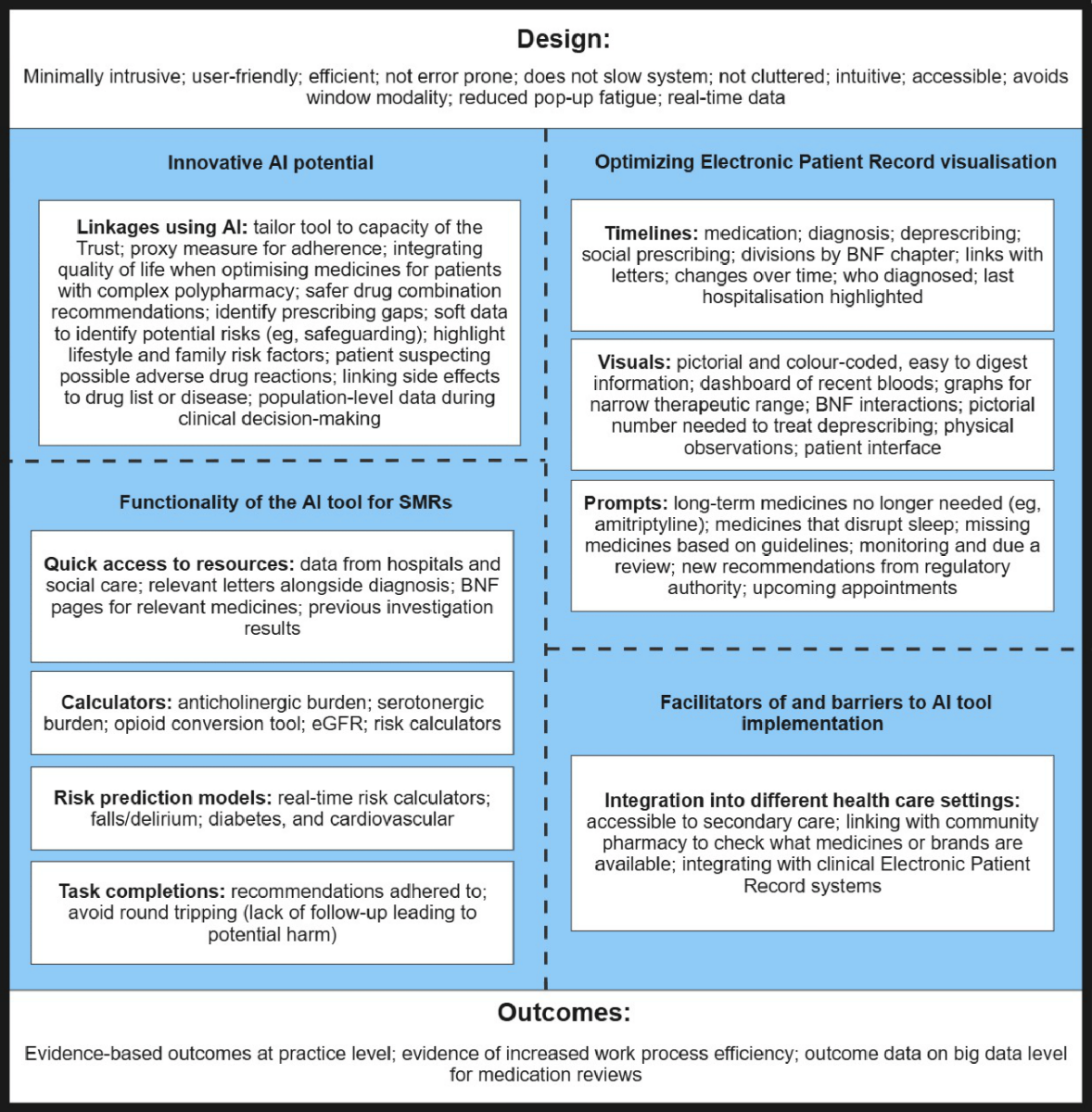
Suggested features for the development of an artificial intelligence (AI)–assisted tool for use in medication reviews. BNF: British National Formulary; eGFR: estimated glomerular filtration rate; SMR: structured medication review.

AI-assisted tools that reduce the time involved in routine tasks, such as finding information, calculating doses, or assessing disease risk, were welcomed. HCPs were conscious of ensuring that any AI-assisted tool did not overwhelm the user with excessive “pop-up” functions on the display and should not overburden the user’s view. There was strong support for a visual timeline that would detail the patient’s diagnosis and prescribing and deprescribing journey, along with relevant investigations, diagnostic letters, which specialty diagnosed the condition and started the medication, and the reasons for certain medicine changes. Figure 2 presents the suggested AI-assisted tool features:

I’ve been dreaming about the timeline you showed [laughs] to be able to, in the way that I’ve imagined it, at the click of a button know...when all the drugs were started and what else was diagnosed around that time [would be] great. And then I don’t have to spend any time trying to put that together, that information is there for me. Thinking outside of a hospital setting, if I’ve got recent bloods and any sort of risk calculations that I want already there on the page from the most recent things, [that is] even better. The amount of time [it would save]; the computer system I work with tells me eGFR, [but] I spend a good chunk of my day calculating everyone’s creatinine clearance.

Participant 2; pharmacist FG1

Participants also described the challenges around investigating the indication for each prescribed medicine, stating that any AI-assisted tool should incorporate the medication indication:

I think for me the most important thing that’s missing is indication-based prescribing. Because when we are doing our medicine reviews just trying to work out why anyone’s on, you know they could be on an ACE inhibitor, why are they on it, you know, they could be on citalopram, you know, why are they on it. And then it’s almost impossible to stop it if you can’t work out why someone started it to begin with. So, I think, for me that would be the key initial thing.

Participant 1; polypharmacy FG

Participants described how information within a patient’s EPR should be visualized, focusing on rearranging and presenting the information in a more intuitive and insightful way to enhance understanding of the patient’s medical journey and provide the information in an easily understandable format to support decision-making. They recommended color-coding blood results, highlighting the most recent hospitalization or medication review, and flagging risky medication combinations. One participant noted the following:

Reviewing the bloods was quite time-consuming at times, but equally, it was color-coded, so if it was in red it means it’s bad news. If it’s not in red, it’s OK. So, a quick eyeball of words can be often sufficient.

Pharmacist 2; interview

Another participant added the following:

The kind of things that we need [are] things like...when was their last medication review done or when was the last SMR done, for example? When was the last hospitalization event, for example? Have they been hospitalized or discharged recently? Then it’d be nice to visualize, you know, and the combinations of drugs which could be risky.

Pharmacist 2; interview

### Functionality of the AI Tool for SMRs

Participants articulated their preferences for the functionality of the AI-assisted tool, particularly emphasizing the need to avoid “pop-up fatigue.” They suggested implementing specific, targeted pop-ups rather than numerous interruptions that could disrupt workflow. One participant remarked as follows:

Any pop ups that you’ve just got to click through does add even seconds to your working day in each patient record so that can be annoying.

Participant 1; GP FG2

Instead, participants preferred notifications for critical issues, such as missing medications in a patient’s record that align with guidelines. For example, a participant stated the following:

If a patient has recently had a myocardial infarction...it pulls out your main groups of drugs from the NICE guidelines and flash up oh they’re not on an ACE inhibitor and you’ve got to say why.

Participant 3; pharmacist FG1

Similarly, they recommended notifications for medications needing review, such as long-term prescriptions of amitriptyline:

Just to make people think, actually. This thing which was started for sleep isn’t helping.

Psychiatrist; interview

Participants also recommended pop-ups for new guideline recommendations:

To meet the Commission of Human Medicines and Medicines and Healthcare products Regulatory Agency recommendations that came in in December, a way of being able to see what antiepileptics have been tried for someone and how long they’ve been on valproate would really inform the review process.

Participant 2; pharmacist FG1

Participants valued AI-assisted tools that enhanced workflow productivity by providing quick access to resources, such as the *British National Formulary* or NICE guidelines based on prescribed medicines or flagged blood test results. One GP noted the following:

I want a dashboard of most recent blood tests and current guidance checked against that for where there’s missing [medication] gaps, just those things alone would probably save about 2- or 3-weeks’ worth of work across my practice per year.

Participant 1; GP FG1

In addition, participants highlighted the need for more efficient access to hospital discharge letters and relevant patient information, which often reside in separate electronic systems, such as EMIS (an EPR system) and Docman (document management system). One participant stated the following:

If you could draw up relevant letters alongside diagnosis so that you don’t have to then trawl through a different system or then write to the GP to request added information on certain diagnosis.

Psychiatrist; interview

Furthermore, automated calculators for clinical decision-making, such as anticholinergic burden calculations, estimated glomerular filtration rate, and opioid conversion calculators, were seen as valuable tools to reduce time and enhance efficiency. For example, a GP shared the following:

I want automatic opioid conversion so that when they’re on tramadol, co-codamol, oramorph, it just gives me a single figure to aim towards and show them, you know, we are at horse tranquilizing level or at dragon tranquilizing level and we need to kind of bring it down.

Participant 1; GP FG1

SMRs, whether proactive or opportunistic, often require multiple appointments and may necessitate further investigations by another HCP in a different setting, such as a hospital. Participants were concerned that current systems might fail to notify HCPs of patients needing follow-up care, potentially leading to harmful outcomes for the patient:

One of the big issues with medical software is not doing what you describe. Computer programmers call it “round tripping,” so basically things aren’t followed up and the loop isn’t closed, and we see huge amounts of error in medicine because of it. For example, we will refer and then it’s off our radar and it’s on someone else’s radar and then they don’t show up at the hospital, and then all of a sudden six months down the line they end up with a malignancy, and then it’s back to the original person who referred problem because that loop wasn’t closed or the data wasn’t followed up. So, having some form of checking system would hugely reduce error and risk within medicine, particularly when it comes to prescribing. So, for example, I’ve referred them to the physio, the physio then closes that loop when they seem them and then that’s dealt with, or the physio does a hydrocortisone injection for example, which I may not need to know about unless I then send them somewhere else, or they are on anticoagulants, or something like that. So, having a tool that ties up those loose ends would be worth its weight in gold I think, particularly in terms of huge amounts of risk reduction.

Participant 1; GP FG1

### Facilitators of and Barriers to AI Tool Implementation

Participants were curious to understand where DynAIRx might be embedded and how it will be adopted in practice. The main goal was to ensure that any new software does not interfere with current work processes. They described concerns about the cost of adopting it in practice, information governance, and potential medico-legal concerns:

I know that I understand X’s point about having it that you are able to record what decisions you made within the tool but I wonder if it might be preferable to have a way of a text summary that could be then copied from the tool into the notes. Or, even a way of having a screen shot into letters so, you know, if there were every any questions what was it that you were looking at on the day what information was the tool giving you and if you then made an interesting decision based off that information, that’s still your decision. But you’ve clearly got what is was that you could see.

Participant 2; pharmacist FG1

Incentivizing the use of the tool to improve uptake was seen as crucial, with participants suggesting that DynAIRx should be offered as an option to HCPs. They recommended explaining the benefits of using DynAIRx as an additional supportive tool alongside traditional EPR systems, providing examples, such as improvements in efficiency, ease of use, and quick access to information. In addition, HCPs recognized that technology is a double-edged sword, which could either reduce or increase the gap between the HCP and the patient. Consequently, HCPs were open to incorporating a patient interface that would present information pictorially to support shared decision-making. Patients indicated that they would find this useful as they were keen to take a more active role in decision-making. However, many patients were unaware of medication review–related services available to help optimize their medicines:

I have never been involved in a review either...Would it just be the GP?...I am going to ask this time whether I can be involved.

Patient 3; patient, multimorbidity FG

HCPs stated that, from their experience, patients are fairly comfortable with technology, which was echoed in the patient FGs:

I have no problem whatsoever with using [AI] tools, especially if it is to back up the thought process.

Patient 4; patient, multimorbidity FG

What I think, being a patient, is that you need to have the trust from the GP and the GP pharmacist, whoever is prescribing and using AI, they need to educate people. They need to raise awareness about it. And then you have the trust with the GP then you feel comfortable.

Patient 1; patient, multimorbidity FG

However, this did not necessarily mean that all patients were able to use technology themselves to communicate or provide feedback to HCPs through surveys and questionnaires, suggesting the need to consider potential digital exclusion:

Not digitally everybody, there are some barriers, language barriers, cultural barriers, and economic barriers for the disadvantaged so we have to think about equity as well. So these barriers are obviously not everything, but we should open up, give the opportunity to all those populations to have the opportunity to discuss and take it rather than have both, it should be both.

Patient; complex multimorbidity FG

While HCPs expressed a desire for risk-prediction tools within the DynAIRx software, some remained skeptical about these tools. They emphasized the importance of providing evidence that the model had been validated or that the software itself improved patient outcomes:

One of the reasons why people may not use it is that they, clinicians often ask for evidence, so they need to see evidence. A published paper or a trial of actually being useful. So does it improve outcomes with patients? These one of the biggest barriers that I’ve come across in my time with tools.

Pharmacist 2; interview

In addition, the need to ensure confidentiality and consent in AI-assisted tool use is paramount, given the amount of data that would be incorporated into DynAIRx. Patients were aware of the fast-paced innovations taking place in technology and the expectation that technology should be included in health care practice:

The world is changing. First we heard about the autopilot, now they are testing [cars] without a driver, virtual GP...now AI in the medical sector as well. The world is changing.

Participant 2; patient

## Discussion

### Principal Findings

Our study outlines the user requirements for the development of an AI-assisted tool to improve the process of conducting an SMR involving patients with complex multimorbidity and polypharmacy. This includes optimizing EPR visualization with a focus on developing patient timelines that outline the patient’s medical journey to include when a condition was diagnosed, associated medicines prescribed for that condition, and any associated laboratory results, to name a few. Moreover, participants described the preference for evidence-based outcomes and the use of explainable AI to identify patients at risk of medication-related harm who would benefit from an SMR, determine their risk trajectory over time, and align those patients to practice staff capacity. By “explainable AI,” we mean models or algorithms designed with transparent logic or post hoc interpretation methods (eg, feature-importance heatmaps and rule-based approximations) that allow HCPs to understand why a particular patient was flagged as high risk rather than relying on opaque “black box” predictions. From our findings, it is clear that HCPs require an AI-assisted tool that will streamline their work processes when conducting an SMR to easily find information related to the patient and incorporate any risk prediction models. Studies show that CDSSs have the potential to improve process outcomes [15,16]; however, access to CDSSs alone does not guarantee user acceptability or uptake [17]. Concerns remain that complex clinical decision-making, particularly for patients with multimorbidity and polypharmacy, may not be easily translated into algorithms [18] or, at the very least, may have HCPs view the validity of algorithms with some skepticism [19]. This can be seen in the literature that reflects HCP preference for knowledge-based CDSSs over non–knowledge-based CDSSs [19,20]. Despite this, studies show that the lack of algorithm complexity in CDSSs can frustrate HCPs, particularly in how information is presented [18,21]. Moreover, user-centered design is crucial in the development of a CDSS to optimize how information is presented to HCPs to manage cognitive load, alert fatigue, and the impact on workflow [22,23].

### Comparisons With Prior Work

AI-assisted tools designed to assist in SMR processes must be tested to ensure they effectively identify both patients who are at risk of medication-related harm and those who have the greatest capacity to benefit from an SMR. These 2 groups are not necessarily synonymous, as some variables that may contribute to harm are not modifiable (eg, very advanced age and frailty), whereas the capacity to benefit from an SMR may be determined by modifiable factors (such as identification of prescribing cascades, drug-drug or drug-disease interactions, and adverse drug reactions). The tool must be sensitive when identifying patients at high risk and display medicines that could be optimized or deprescribed efficiently, enabling future evaluation of medication-related interventions using available data. Patients taking part in an SMR are also likely to have multiple appointments and be referred to different specialists, making it difficult for the HCPs who initiated the SMR to follow this journey. Few studies have explored the interoperability between primary and secondary health care settings, which is essential for effective communication and coordination between HCPs, and is perceived to have a high impact on patient safety [24,25]. Our findings indicate a clear need for an AI-assisted tool to support clinicians and patients during consultations. An integrated system within the EPR software could help summarize and visualize patient journeys and medicine-related information, present personalized risks of harms and benefits of medicines combinations [26], highlight the uncertainties in these risks, and support shared decision-making with patients. While the findings of this study are based on anticipated rather than observed experiences with an AI-assisted tool, engaging end users at the outset before tool development enables the design of an AI-assisted tool that is contextually relevant, cost-effective, and more likely to be adopted in practice [27,28]. Future stages of this work will involve the development and testing of a prototype to validate and refine these preliminary findings through direct user interaction and usability testing, as demonstrated by previous studies [29-32]. This iterative, user-centered process will help ensure that the AI-assisted tool is not only functional but also implementable in real-world settings.

Previous literature highlights the preference among HCPs for knowledge-based over non–knowledge-based CDSSs, which aligns with concerns about the validity and acceptability of algorithms in complex clinical scenarios [19,20]. While frustrations with simplistic CDSS algorithms have been noted [14], our study contributes additional evidence emphasizing the need for user-centered design to address cognitive load, alert fatigue, and workflow impact. The clinical decision support five rights model states that the right information should be presented to the right person, in the right format, via the right channel, and at the right time [16]. Studies on the development of CDSSs emphasize the importance of understanding the needs of users and receivers during the early stages of software development [17,18]. Interoperability between health care settings, while unexplored in previous work, emerged as a critical component for effective SMR implementation and improved patient safety in this study [10,19]. Integrated care boards link primary and secondary care data across their local areas with input from EPR vendors, such as EMIS and SystmOne. An approach consisting of fully linked local data, with minor regional differences in data formatting, may begin to emerge in the coming years as one way to proceed. Efforts, such as the National Health Service Federated Data Platform, aim to unify these actions across the nation and may provide additional clarity on this approach in the coming years. The next stage of the DynAIRx project will involve learning from the user requirements of our stakeholder groups to develop prototypes of an AI-assisted tool. We anticipate the developed prototypes to likely fall under the category of a knowledge support system, which provides HCPs with knowledge that already exists, such as contextual information about a patient drawn from several sources, including historic data on clinical outcomes from comparable patient groups, medical knowledge, and AI to support HCPs during consultations [29,33,34].

To bridge our user-centered requirements with real-world adoption, we recommend framing the development and deployment of the AI-assisted tool for SMRs within established implementation science frameworks, such as the Consolidated Framework for Implementation Research [35]. Under the Consolidated Framework for Implementation Research, the intervention characteristics (eg, usability, adaptability of the patient-timeline visualization, and risk-prediction algorithms), the inner setting (practice culture and EPR interoperability), the outer setting (national data-linkage initiatives, such as the National Health Service Federated Data Platform), the characteristics of individuals (clinician attitudes toward explainable AI), and the implementation process (engagement, training, and feedback loops) all warrant deliberate planning and tailoring.

A staged implementation approach could proceed as presented in Textbox 1.

**Staged implementation approach.**

**Before implementation (exploration and preparation)**

- Conduct targeted workflow analyses in a small number of pilot practices to refine how the timeline and risk outputs map onto existing structured medication review processes
- Engage local electronic patient record vendors (eg, EMIS and SystmOne) to configure interoperability and data-security protocols

**Implementation (initial rollout and training)**

- Deploy the prototype in 3 to 5 “early adopter” practices, offering hands-on workshops and “superuser” support
- Establish a feedback channel (eg, biweekly focus groups) to iteratively refine

**Sustainment (scale-up and longitudinal support)**

- Expand to additional practices, embedding the tool within organizational reporting cycles
- Integrate decision-support outputs into routine safety audits and continuing professional development activities

Building on our staged implementation plan, future studies should focus on prospective validation of the AI-assisted tool for SMRs in live clinical settings. Key next steps include the following:

- Pilot effectiveness trials—randomized or stepped-wedge designs comparing standard SMR to SMR with AI support, measuring both process (eg, time per review and alert response rates) and patient-level outcomes (eg, incidence of medication-related harm)
- Usability and acceptability—mixed methods evaluations combining system-log analytics with qualitative interviews to understand how clinicians interact with features such as patient timelines and risk predictions
- Subgroup analyses—examining performance across different patient demographics and varying levels of multimorbidity complexity to ensure equitable benefit

### Limitations

This research is part of a larger qualitative study exploring the barriers to SMRs and potential AI-assisted tools. Given the focus on digital-driven solutions, the HCP participants likely included those with a particular interest in such innovations, although efforts were made to include a diverse range of HCPs from different practice backgrounds, regions, and care settings to mitigate this bias and help strengthen the generalizability of our findings within the context of SMR practices. Moreover, our eligibility criteria focused on recruiting HCPs who are actively involved in conducting medication reviews to explore the barriers to efficient and effective SMRs and how AI-assisted tools may address these barriers [4]. Some FGs had fewer participants due to the competing demands on clinicians’ time. However, the data collected were rich and contributed significantly to achieving thematic saturation. While this study provides valuable insights into the user requirements for developing an AI-assisted tool, certain limitations should be acknowledged. First, the diversity of the patient group included in this study may not fully represent the broader patient population, particularly in terms of familiarity with AI-assisted tools. Patients’ knowledge and understanding of such systems may vary significantly, potentially influencing the feedback provided. As a result, the findings may not fully capture the perspectives of patients with lower levels of digital health literacy or those who have more experience engaging with AI-assisted tools in clinical settings. Second, the study captures participants’ hypothetical perceptions of the AI-assisted tool rather than their experiences of real-world implementation and use. While the findings highlight anticipated benefits and potential facilitators, they may not fully reflect the complexities of adoption and sustained use in practice. Consequently, the research team will develop a prototype based on our findings, which will be tested to validate results from this study and refine and iterate the prototype through a series of think-aloud sessions and semistructured interviews with HCPs. This will ensure the prototype is developed based on user requirements and allow the user to explore the utility of the tool. Moreover, we anticipate a future evaluation of the AI-assisted tool by implementing it into the HCPs’ routine workflow. Future research involving live system implementation and longitudinal evaluation would be valuable in assessing the feasibility and actual impact of AI-assisted tool integration in health care settings.

### Conclusions

This study highlights the potential of AI-assisted tools to enhance the SMR process for patients with complex multimorbidity and polypharmacy and the user requirements to develop an AI-assisted tool. AI-assisted tools may have the potential to improve patient identification for an SMR, assist the HCP to optimize patient medication by optimizing the EPR visualization of the patients’ medical and social history, and support shared decision-making between HCPs and patients. In order to realize the full potential of AI-assisted tools for SMRs, national and local policy makers should consider earmarking targeted funding streams, such as through the National Health Service Digital’s Innovation Accelerator or PCN transformation budgets, to subsidize early implementation and integration with EPR systems. Embedding AI-SMR competencies into continuing professional development requirements for pharmacists and GPs, alongside dedicated training grants, will help build workforce capability and ensure equitable uptake. Finally, linking reimbursement incentives (eg, enhanced quality and outcomes framework points to multimorbidity reviews that use validated AI support) could further drive adoption and standardize best practice across PCNs. However, to ensure successful implementation, it is essential to address concerns, such as cognitive load, alert fatigue, and system interoperability. The findings underscore the importance of developing explainable, evidence-backed tools that align with clinical workflows and demonstrate clear benefits to patient outcomes. Ultimately, integrating such tools into an EPR has the potential to improve the efficiency and effectiveness of SMRs, benefiting both patients and health care systems.

## Appendix

Multimedia Appendix 1 COREQ checklist.

## Abbreviations

AI: artificial intelligence
CDSS: clinical decision support system
COREQ: Consolidated Criteria for Reporting Qualitative Research
DynAIRx: artificial intelligence for dynamic prescribing optimization and care integration in multimorbidity
EPR: electronic patient record
FG: focus group
GP: general practitioner
HCP: health care professional
LLM: large language model
ML: machine learning
NICE: National Institute for Health and Care Excellence
PCN: primary care network
SMR: structured medication review

## References

[1] Network contract directed enhanced service: structured medication reviews and medicines optimisation: guidance. National Health Service England.

[2] Network contract DES guidance for 2024/25: part A - clinical and support services (Section 8). National Health Service England.

[3] Network contract directed enhanced service – contract specification 2021/22 – PCN requirements and entitlements. National Health Service England.

[4] Abuzour AS, Wilson SA, Woodall AA, Mair FS, Clegg A, Shantsila E, Gabbay M, Abaho M, Aslam A, Bollegala D, Cant H, Griffiths A, Hama L, Leeming G, Lo E, Maskell S, O'Connell M, Popoola O, Relton S, Ruddle RA, Schofield P, Sperrin M, Staa TV, Buchan I, Walker LE (2024). A qualitative exploration of barriers to efficient and effective structured medication reviews in primary care: findings from the DynAIRx study. PLoS One.

[5] The leaders in providing EMIS web and SystmOne templates and resources. Ardens Healthcare Informatics.

[6] A health management tool for people with diabetes co-created by Lilly and NHS Devon CCG. PARM Diabetes.

[7] Abuzour AS, Magola-Makina E, Dunlop J, O'Brien A, Khawagi WY, Ashcroft DM, Brown P, Keers RN (2022). Implementing prescribing safety indicators in prisons: a mixed methods study. Br J Clin Pharmacol.

[8] Rodgers S, Taylor AC, Roberts SA, Allen T, Ashcroft DM, Barrett J, Boyd MJ, Elliott RA, Khunti K, Sheikh A, Laparidou D, Siriwardena AN, Avery AJ (2022). Scaling-up a pharmacist-led information technology intervention (PINCER) to reduce hazardous prescribing in general practices: multiple interrupted time series study. PLoS Med.

[9] Ivers N, Jamtvedt G, Flottorp S, Young JM, Odgaard-Jensen J, French SD, O'Brien MA, Johansen M, Grimshaw J, Oxman AD (2012). Audit and feedback: effects on professional practice and healthcare outcomes. Cochrane Database Syst Rev.

[10] Rao A, Kim J, Kamineni M, Pang M, Lie W, Dreyer KJ, Succi MD (2023). Evaluating GPT as an adjunct for radiologic decision making: GPT-4 versus GPT-3.5 in a breast imaging pilot. J Am Coll Radiol.

[11] Kobraei K, Baradaran M, Sadeghi SM, Masumshah R, Eslahchi C ADEP: a novel approach based on discriminator-enhanced encoder-decoder architecture for accurate prediction of adverse effects in polypharmacy. arXiv. Preprint posted online on May 31, 2024.

[12] Ong JC, Jin L, Elangovan K, Lim GY, Lim DY, Sng GG, Ke Y, Tung JY, Zhong RJ, Koh CM, Lee KZ Development and testing of a novel large language model-based clinical decision support systems for medication safety in 12 clinical specialties. arXiv. Preprint posted online on January, 2024.

[13] Tapper J (2024). DrugGPT: new AI tool could help doctors prescribe medicine in England. The Guardian.

[14] Liu F, Zhou H, Zhang W, Huang G, Clifton L, Eyre D, Luo H, Liu F, Branson K, Schwab P, Wu X, Zheng Y RETRACTED: DrugGPT: a knowledge-grounded collaborative large language model for evidence-based drug analysis. Research Square. Preprint posted online on October 6, 2023.

[15] Bright TJ, Wong A, Dhurjati R, Bristow E, Bastian L, Coeytaux RR, Samsa G, Hasselblad V, Williams JW, Musty MD, Wing L, Kendrick AS, Sanders GD, Lobach D (2012). Effect of clinical decision-support systems: a systematic review. Ann Intern Med.

[16] Kwan JL, Lo L, Ferguson J, Goldberg H, Diaz-Martinez JP, Tomlinson G, Grimshaw JM, Shojania KG (2020). Computerised clinical decision support systems and absolute improvements in care: meta-analysis of controlled clinical trials. BMJ.

[17] Kouri A, Yamada J, Lam Shin Cheung J, Van de Velde S, Gupta S (2022). Do providers use computerized clinical decision support systems? A systematic review and meta-regression of clinical decision support uptake. Implement Sci.

[18] Greenes RA, Bates DW, Kawamoto K, Middleton B, Osheroff J, Shahar Y (2018). Clinical decision support models and frameworks: seeking to address research issues underlying implementation successes and failures. J Biomed Inform.

[19] Meunier PY, Raynaud C, Guimaraes E, Gueyffier F, Letrilliart L (2023). Barriers and facilitators to the use of clinical decision support systems in primary care: a mixed-methods systematic review. Ann Fam Med.

[20] Kim SY, Kim DH, Kim MJ, Ko HJ, Jeong OR (2024). XAI-based clinical decision support systems: a systematic review. Appl Sci.

[21] Sirajuddin AM, Osheroff JA, Sittig DF, Chuo J, Velasco F, Collins DA (2009). Implementation pearls from a new guidebook on improving medication use and outcomes with clinical decision support. Effective CDS is essential for addressing healthcare performance improvement imperatives. J Healthc Inf Manag.

[22] Kilsdonk E, Peute LW, Jaspers MW (2017). Factors influencing implementation success of guideline-based clinical decision support systems: a systematic review and gaps analysis. Int J Med Inform.

[23] Van de Velde S, Heselmans A, Delvaux N, Brandt L, Marco-Ruiz L, Spitaels D, Cloetens H, Kortteisto T, Roshanov P, Kunnamo I, Aertgeerts B, Vandvik PO, Flottorp S (2018). A systematic review of trials evaluating success factors of interventions with computerised clinical decision support. Implement Sci.

[24] Martínez-García A, Moreno-Conde A, Jódar-Sánchez F, Leal S, Parra C (2013). Sharing clinical decisions for multimorbidity case management using social network and open-source tools. J Biomed Inform.

[25] Fraccaro P, Arguello Casteleiro M, Ainsworth J, Buchan I (2015). Adoption of clinical decision support in multimorbidity: a systematic review. JMIR Med Inform.

[26] Fahmi A, Wong D, Walker L, Buchan I, Pirmohamed M, Sharma A, Cant H, Ashcroft DM, van Staa TP (2023). Combinations of medicines in patients with polypharmacy aged 65-100 in primary care: large variability in risks of adverse drug related and emergency hospital admissions. PLoS One.

[27] Greenhalgh T, Wherton J, Papoutsi C, Lynch J, Hughes G, A'Court C, Hinder S, Fahy N, Procter R, Shaw S (2017). Beyond adoption: a new framework for theorizing and evaluating nonadoption, abandonment, and challenges to the scale-up, spread, and sustainability of health and care technologies. J Med Internet Res.

[28] MAGUIRE M (2001). Methods to support human-centred design. Int J Hum Comput Stud.

[29] van Staa T, Sharma A, Palin V, Fahmi A, Cant H, Zhong X, Jury F, Gold N, Welfare W, Ashcroft D, Tsang JY, Elliott RA, Sutton C, Armitage C, Couch P, Moulton G, Tempest E, Buchan IE (2023). Knowledge support for optimising antibiotic prescribing for common infections in general practices: evaluation of the effectiveness of periodic feedback, decision support during consultations and peer comparisons in a cluster randomised trial (BRIT2) - study protocol. BMJ Open.

[30] Yuan MJ, Finley GM, Long J, Mills C, Johnson RK (2013). Evaluation of user interface and workflow design of a bedside nursing clinical decision support system. Interact J Med Res.

[31] Blanes-Selva V, Asensio-Cuesta S, Doñate-Martínez A, Pereira Mesquita F, García-Gómez JM (2023). User-centred design of a clinical decision support system for palliative care: insights from healthcare professionals. Digit Health.

[32] Larsen K, Akindele B, Head H, Evans R, Mehta P, Hlatky Q, Krause B, Chen S, King D (2022). Developing a user-centered digital clinical decision support app for evidence-based medication recommendations for type 2 diabetes mellitus: prototype user testing and validation study. JMIR Hum Factors.

[33] Hurley R, Jury F, van Staa TP, Palin V, Armitage CJ (2023). Clinician acceptability of an antibiotic prescribing knowledge support system for primary care: a mixed-method evaluation of features and context. BMC Health Serv Res.

[34] Rydberg EM, Insulan J, Rolfson O, Mohaddes M, Ahlstrom L (2022). Knowledge support for ankle fractures in the Swedish Fracture Register - a qualitative study of physicians' experiences. BMC Health Serv Res.

[35] Damschroder LJ, Reardon CM, Widerquist MA, Lowery J (2022). The updated Consolidated Framework for implementation research based on user feedback. Implement Sci.

