# Supplementary material for: Developing an AI-Assisted Tool That Identifies Patients With Multimorbidity and Complex Polypharmacy to Improve the Process of Medication Reviews: Qualitative Interview and Focus Group Study
Source: J Med Internet Res. 2026 Jan 8;28:e74304. doi: 10.2196/74304 (PMC12828319; doi:10.2196/74304)
Supplement: Multimedia Appendix 1 [file jmir_v28i1e74304_app1.pdf]

Consolidated criteria for reporting qualitative studies (COREQ): 32-item checklist

No. Item	Guide questions/description	Reported on Page #
**Domain 1: Research team and reflexivity**		
Personal Characteristics		
1. Inter viewer/facilitator	Which author/s conducted the interview or focus group?	5
2. Credentials	What were the researcher's credentials? E.g. PhD, MD	1
3. Occupation	What was their occupation at the time of the study?	1
4. Gender	Was the researcher male or female?	Both male and female
5. Experience and training	What experience or training did the researcher have?	4-5
Relationship with participants		
6. Relationship established	Was a relationship established prior to study commencement?	5-6
7. Participant knowledge of the interviewer	What did the participants know about the researcher? e.g. personal goals, reasons for doing the research	5-6
8. Interviewer characteristics	What characteristics were reported about the inter viewer/facilitator? e.g. Bias, assumptions, reasons and interests in the research topic	5-6

**Domain 2: study design**		
Theoretical framework		
9. Methodological orientation and Theory	What methodological orientation was stated to underpin the study? e.g. grounded theory, discourse analysis, ethnography, phenomenology, content analysis	5-6
Participant selection		
10. Sampling	How were participants selected? e.g. purposive, convenience, consecutive, snowball	5-6
11. Method of approach	How were participants approached? e.g. face-to-face, telephone, mail, email	5-6
12. Sample size	How many participants were in the study?	7-8
13. Non-participation	How many people refused to participate or dropped out? Reasons?	7
Setting		
14. Setting of data collection	Where was the data collected? e.g. home, clinic, workplace	5-6
15. Presence of non-participants	Was anyone else present besides the participants and researchers?	5-6
16. Description of sample	What are the important characteristics of the sample? e.g. demographic data, date	7-8
Data collection		
17. Interview guide	Were questions, prompts, guides provided by the authors? Was it pilot tested?	5-6
18. Repeat interviews	Were repeat interviews carried out? If yes, how many?	No
19. Audio/visual recording	Did the research use audio or visual recording to collect the data?	6-7
20. Field notes	Were field notes made during and/or after the interview or focus group?	6-7
21. Duration	What was the duration of the interviews or focus group?	6
22. Data saturation	Was data saturation discussed?	7
23. Transcripts returned	Were transcripts returned to participants for comment and/or correction?	No
**Domain 3: analysis and findings**		
Data analysis		

24. Number of data coders	How many data coders coded the data?	7
25. Description of the coding tree	Did authors provide a description of the coding tree?	No
26. Derivation of themes	Were themes identified in advance or derived from the data?	7
27. Software	What software, if applicable, was used to manage the data?	7
28. Participant checking	Did participants provide feedback on the findings?	No
Reporting		
29. Quotations presented	Were participant quotations presented to illustrate the themes/findings? Was each quotation identified? e.g. participant number	Yes
30. Data and findings consistent	Was there consistency between the data presented and the findings?	Yes
31. Clarity of major themes	Were major themes clearly presented in the findings?	Yes
32. Clarity of minor themes	Is there a description of diverse cases or discussion of minor themes?	Yes
